# A Novel Genetic Score Approach Using Instruments to Investigate Interactions between Pathways and Environment: Application to Air Pollution

**DOI:** 10.1371/journal.pone.0096000

**Published:** 2014-04-22

**Authors:** Marie-Abele Bind, Brent Coull, Helen Suh, Robert Wright, Andrea Baccarelli, Pantel Vokonas, Joel Schwartz

**Affiliations:** 1 Department of Environmental Health, Harvard School of Public Health, Boston, Massachusetts, United States of America; 2 Department of Biostatistics, Harvard School of Public Health, Boston, Massachusetts, United States of America; 3 Department of Epidemiology, Harvard School of Public Health, Boston, Massachusetts, United States of America; 4 VA Normative Aging Study, Boston University School of Medicine, Boston, Massachusetts, United States of America; Maastricht University Medical Center, Netherlands

## Abstract

Air pollution has been associated with increased systemic inflammation markers. We developed a new pathway analysis approach to investigate whether gene variants within relevant pathways (oxidative stress, endothelial function, and metal processing) modified the association between particulate air pollution and fibrinogen, C-reactive protein (CRP), intercellular adhesion molecule-1 (ICAM-1), and vascular cell adhesion molecule-1 (VCAM-1). Our study population consisted of 822 elderly participants of the Normative Aging Study (1999–2011). To investigate the role of biological mechanisms and to reduce the number of comparisons in the analysis, we created pathway-specific scores using gene variants related to each pathway. To select the most appropriate gene variants, we used the least absolute shrinkage and selection operator (Lasso) to relate independent outcomes representative of each pathway (8-hydroxydeoxyguanosine for oxidative stress, augmentation index for endothelial function, and patella lead for metal processing) to gene variants. A high genetic score corresponds to a higher allelic risk profile. We fit mixed-effects models to examine modification by the genetic score of the weekly air pollution association with the outcome. Among participants with higher genetic scores within the oxidative stress pathway, we observed significant associations between particle number and fibrinogen, while we did not find any association among participants with lower scores (p_interaction_ = 0.04). Compared to individuals with low genetic scores of metal processing gene variants, participants with higher scores had greater effects of particle number on fibrinogen (p_interaction_ = 0.12), CRP (p_interaction_ = 0.02), and ICAM-1 (p_interaction_ = 0.08). This two-stage penalization method is easy to implement and can be used for large-scale genetic applications.

## Introduction

To better understand molecular mechanisms, one can investigate the role of gene variants individually or collectively within a biological pathway. Pathway analysis may be more informative of the underlying biology. Mechanisms by which particulate air pollution is linked to exacerbation of cardiovascular disease (CVD) morbidity and mortality are not fully understood [1]. Previous studies have suggested biological changes after air pollution exposure such as thrombosis [2], systemic cytokine-mediated inflammation [3], and impaired endothelial function [4], [5], especially among the elderly.

To better understand the molecular mechanisms linking air pollution and CVD, we chose to examine the role gene variants play in modifying the adverse air pollution effects by considering genetic pathway-air pollution interactions [6], since polymorphisms in pathways unrelated to how exposure produces response are unlikely to modify the exposure-response relation. Oxidative stress, endothelial dysfunction, and impaired metal processing are potential intermediate biological responses that may relate air pollution to CVD [6]–[9]. The oxidative stress pathway has been shown to be central to both the toxicology of air pollution and the pathogenesis of coronary atherosclerosis [10]–[12]. The endothelial dysfunction pathway has also been identified as a key player in air pollution molecular responses [8], [13]. Metals in particles have been associated with increased oxidative stress and inflammation, and this was reduced in knockout mice deficient in metal transport mechanisms. In addition, exposure to metals on particles has been associated with decreases in heart rate variability [7], [14], [15] and whole-blood coagulation time [16]–[19]. We therefore hypothesized that gene variants related to metal processing play an important role in biological responses to air pollutants having metals attached to them.

This study builds upon previous research by investigating three potential pathways (oxidative stress, impaired endothelial function, and metal processing dysfunction) that may modify the association between air pollution and the outcomes fibrinogen, C-reactive protein, intercellular adhesion molecule-1 (ICAM-1) and vascular cell adhesion molecule-1 (VCAM-1), which have been related to coronary heart disease and atherosclerosis [20], [21]. We developed a new allelic score method following an existing parsimonious approach that groups gene variants related to similar molecular mechanisms [22]. The study focused on the elderly, a potentially susceptible subgroup.

## Materials

### Study population

This study included participants from the Normative Aging Study (NAS), a prospective cohort of aging established in 1963, enrolling men from the Greater Boston area. Participants underwent medical examinations every three to five years. More detailed longitudinal characteristics on the study population can be found elsewhere [23]. We obtained one to five measurements of fibrinogen, C-reactive protein, ICAM-1, and VCAM-1 on 822 participants between 1999 and 2011.

### Ethics statement

This study was approved by the Harvard School of Public Health and the Veteran Administration Institution Review Boards (IRB). Participants provided written informed consent to participate in this study, which was approved by the Veteran Administration IRB.

### Air pollution assessment

We measured particulate concentrations at the Harvard supersite located 1 km from the examination site. We measured hourly particle number per cm^3^ (0.007–3 µm) with a Condensation Particle Counter (TSI Inc, Model 3022A, Shoreview, MN), hourly ambient fine particle (PM_2.5_) concentrations with a Tapered Element Oscillation Microbalance (Model 1400A, Rupprecht and Pastashnick, East Greenbush, NY), and hourly PM_2.5_ black carbon concentrations using an Aethalometer (Magee Scientific Co., Model AE-16, Berkeley, CA).

The relevant exposure window for gene-air pollution interactions on coagulation, and inflammation is unclear. Results of previous studies suggested an association between air pollution and blood markers of inflammation and coagulation spread over several days and weeks prior to the time of assessment [5], [23]. To limit the number of statistical comparisons, we *a priori* considered only concentrations averaged over the week preceding each participant's examination.

### Genotyping assessment

An exhaustive list of the genes we considered can be found in Tables S1 to S3 in File S1. We related each gene to one of the three pathways (oxidative stress, impaired endothelial function, and metal processing dysfunction) based on their biological functionality provided *GeneCards* [24]. A brief description of each gene's function can be found in Tables S4 to S6 in File S1. We coded gene variants as follows: 0 for wild type, 1 for heterozygous, and 2 for homozygous; except for glutathione S-transferase mu 1 (GSTM1) and glutathione S-transferase theta 1 (GSTT1) that were coded 0 (deletion) or 1 (no deletion).

Assays were genotyped using the Sequenom MassArray MALDI-TOF Mass Spectrometer with semi-automated primer design and implementation of the short extension method (San Diego, CA). The MassARRAY System offers several features including very high sensitivity, capacity to analyze multiple classes of genetic markers, ability to quickly create and modify customized assay panels, flexible sample throughput (from few to thousands), and high quality data with low operating costs. Multiplexed polymerase chain reaction assays were designed using the Sequenom Spectro Designer software (San Diego, CA).

### Blood marker outcomes assessment

At each visit, we measured concentrations of plasma fibrinogen using MDA Fibriquick (Trinity Biotech, Bray, Ireland), a bovine thrombin reagent (Clauss, reagent number 17). We quantified C-reactive protein levels using an immunoturbidimetric assay on the Hitachi 917 analyzer (Roche Diagnostics Indianapolis, IN). We assessed plasma ICAM-1 and VCAM-1 concentrations with an enzyme-linked immunoabsorbent assay method (R&D Systems, Minneapolis, MN). This technique has been exhaustively tested for superior quality and reproducibility.

## Methods

### 1^st^-stage: Gene variants selection using the least absolute shrinkage and selection operator (Lasso)

We related gene variants to each pathway based on their functionality. Because of the large number of gene variants for each gene and within each pathway, we chose to penalize them using the Lasso method, which simultaneously performs variables selection and regression coefficients estimation [25]. To select the relevant gene variants, we considered the following outcomes (not being examined for air pollution) for each pathway: 8-hydroxydeoxyguanosine (8-ohdg) for oxidative stress, augmentation index for endothelial function, and patella lead for metal processing. We included gene variants following dominant models ({0} versus {1 or 2}), recessive ({0 or 1} versus {2}), deletions ({0} versus {1}), and adjusted for age. The regression model was:
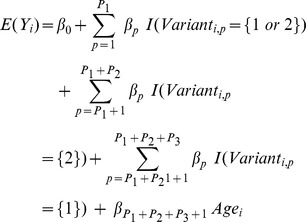





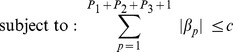
where *Yi* corresponds to levels of 8-ohdg, augmentation index, and patella lead levels for each participant *i* and *c* is a non-negative tuning parameter. P_1_ and P_2_ represent the numbers of gene variants that could follow a dominant and recessive model, respectively. P_3_ corresponds to the number of gene variants that were deletions. The Lasso problem can be rewritten in the equivalent Lagrangian form in which the regression coefficients solved:



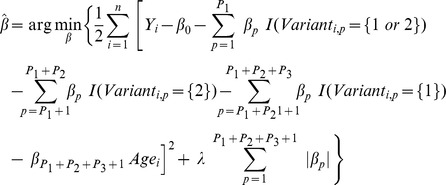
where *λ* is a non-negative parameter that controls the amount of shrinkage: the larger the value of *λ*, the greater the amount of shrinkage. There is a one-to-one correspondence between *λ* and *c*. The latter constraint (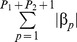) makes the solutions nonlinear in the *Y_i_* and computing the Lasso solution is a quadratic programming problem. We chose the penalty term of the Lasso regression (*λ*) based on the lowest Akaike Information Criterion (AIC). This approach created a reduced set of candidate gene variants related to each pathway. We adjusted for age at this stage to explain some variability of the instrumental outcomes, and thus to help the selection process to be more accurate. We conducted the analysis using the Least Angle Regression (“lars”) R package [26].

### 2^nd^-stage: Genetic-score construction

Using the signs of the non-zero coefficients of the Lasso penalization, we summed up gene variants related to each pathway to construct three score variables representing the allelic profile of each participant. The score for each individual *i* was:
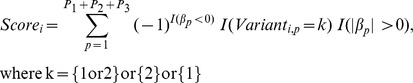



### 3^rd^-stage: Genetic-pathway-air pollution interactions

We then dichotomized these score variables using the median of each score's distribution and used these resulted binary variables to investigate effect modification. A high score corresponds to a higher allelic risk profile. Another score approach has been used successfully before to examine gene-environment by pathway interactions [22]. Fibrinogen, C-reactive protein, ICAM-1, and VCAM-1 were log-transformed to generate a more normal distribution in the residuals. Because many participants had their blood markers measured more than once, we fit separate linear mixed-effects models with a random intercept for each participant. Regression models included the air pollutant, seasonal sine and cosine, temperature, relative humidity, age, body mass index, diabetes, smoking status, statin use, the binary genetic score, and the interaction between the binary genetic score and the air pollutant. At this stage, we still need to control for age because it is a still potential confounding variable.

### Sensitivity analysis

Because our analysis involved 9 tests (n_Tests_ = 3 exposures *3 pathways), we conducted some multiple testing corrections using Bonferroni adjustment.

## Results

### Descriptive statistics

At baseline, the median age of the study participants was 72 years (Table 1). About 14% were diabetics and 37% were statin users. More detailed longitudinal characteristics of the study population can be found elsewhere [23]. Some summary statistics of the air pollutant concentrations are presented in Table 2. PM_2.5_ and black carbon were significantly correlated (correlation coefficient of 0.72).

**Table 1:** Baseline characteristics of the 882 Normative Aging Study participants.

	Mean ± SD	5%	50%	95%	
**Age (years)**	72.4±6.9	62	72	84	
**BMI (kg/m^2^)**	28.3±4.1	22.5	27.8	35.2	
**Fibrinogen** *	350±88.2	239	334	529	
**CRP** **	3.1±5.7	0.3	1.7	9.0	
**ICAM-1** ***	298±101.8	188	288	433	
**VCAM-1** ***	1050±356.1	634	988	1622	
	**N (%)**				
**Diabetes**	113 (14%)				
**Smoking**					
**-Never**	233 (28%)				
**-Current**	37 (67%)				
**-Former**	552 (5%)				
**Statin use**	296 (36%)				

*mg/dL.

**mg/L.

***ng/mL.

**Table 2:** Distribution and correlation between air pollutants for one-week averaged concentrations between 1999 and 2011.

Pollutants' distributions			Mean	25^th^ percentile	Median		75^th^ percentile	
Particle Number (count/cm^3^)			20238	12405	17388		27009	
Black Carbon (µg/m^3^)			0.96	0.65	0.87		1.18	
PM_2.5_ (µg/m^3^)			10.83	7.99	10.12		12.78	
**Spearman correlations between pollutants pairs**								
		Particle number		Black carbon		PM_2.5_		
Particle number		1		0.04		0.12*		
Black carbon				1		0.72*		
PM_2.5_						1		

* p-value <0.05.

### Genetic scores

Among the 18 gene variants candidates for the oxidative stress pathway, seven were chosen by the Lasso procedure: *rs1001179* (+), *rs480575* (−), *rs2071746* (+), *rs5995098* (+), *rs1800566* (+), *rs2282679* (−), and *rs3170633* (−). (+) and (−) represent the sign we used when we added these seven variants up to create the genetic score. Among the nine gene variants candidates for the endothelial function pathway, the Lasso approach chose four of them: *rs12944039* (+), *rs2072324* (+), *rs2255929* (+), and *rs1137933* (−). Similarly, among the nine gene variants candidates for the metal processing pathway, five were chosen by the Lasso penalization *rs224572* (−), *rs422982* (+), *rs12227734* (−), *rs1005559* (−), and *rs1799945* (−). Tables S1 to S3 in File S1 present the *rs* numbers and their corresponding genes.

### Gene-air pollution interactions by pathways

**Figure 1:**
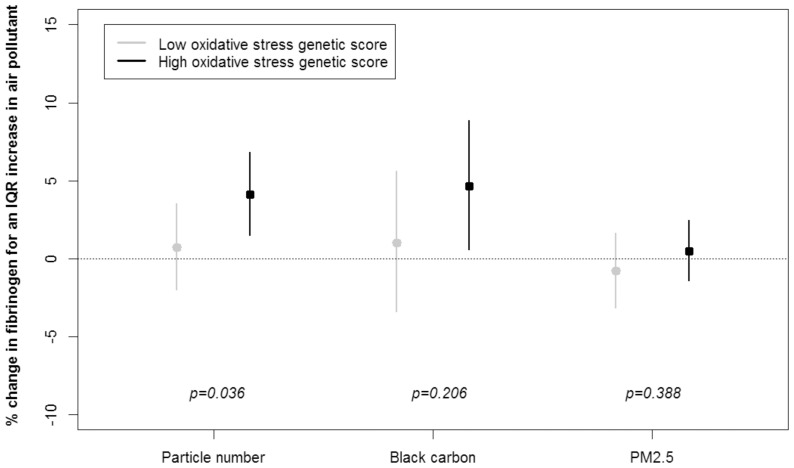
Percent change in fibrinogen for one interquartile range increase in air pollutant according to the oxidative stress genetic score (low versus high).

**Figure 2:**
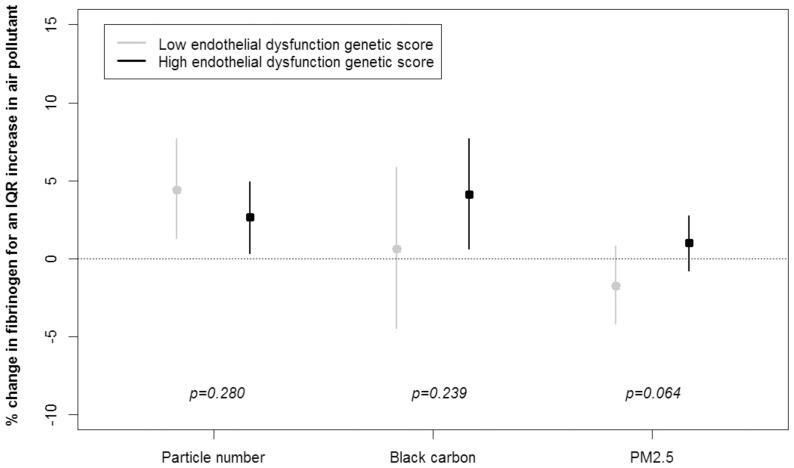
Percent change in fibrinogen for one interquartile range increase in air pollutant according to the endothelial dysfunction genetic score (low versus high).

**Figure 3:**
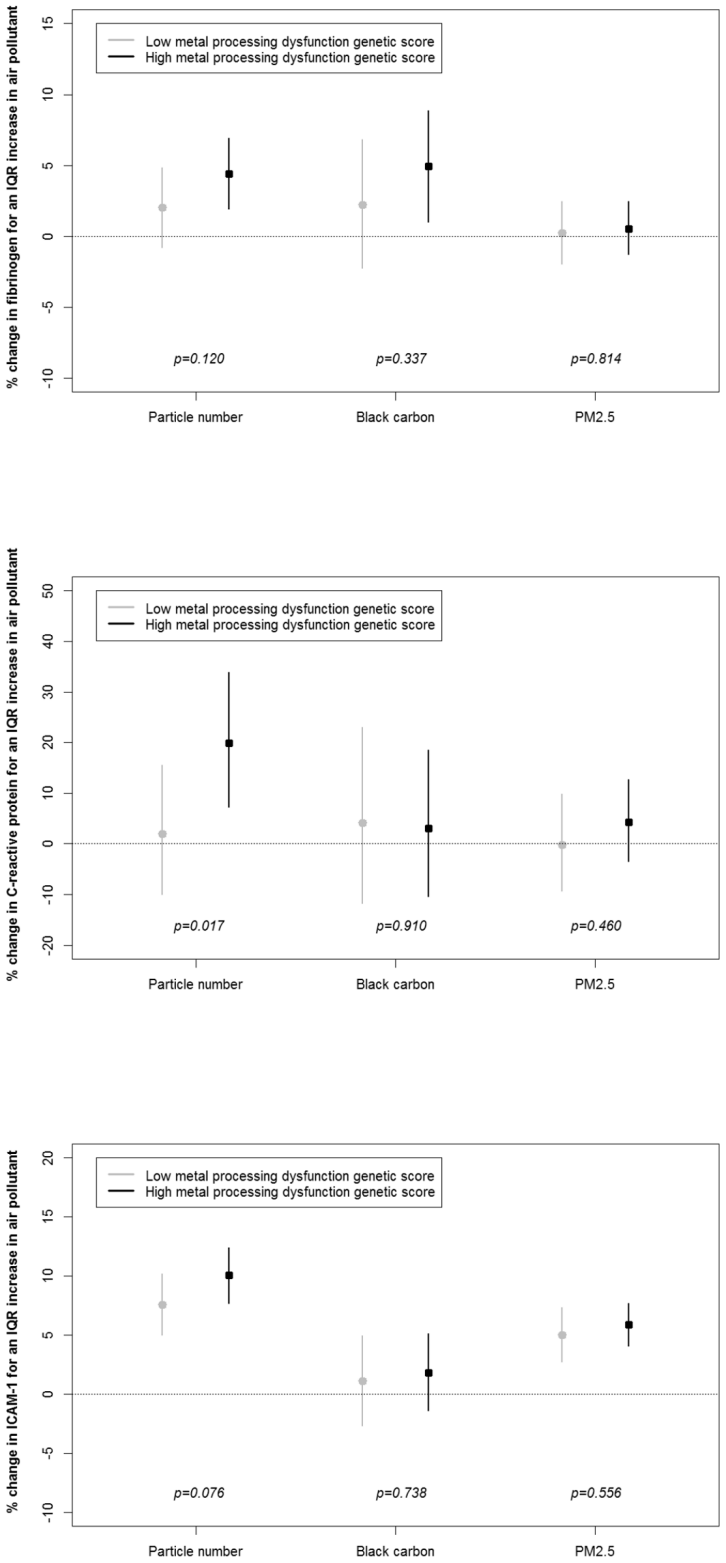
Percent change in blood markers (fibrinogen, C-reactive protein, and ICAM-1) for one interquartile range increase in air pollutant according to metal processing dysfunction genetic score (low versus high).

We observed significant genetic pathway-air pollution interactions on fibrinogen, C-reactive protein, and ICAM-1. We found that the effect of particle number on fibrinogen was modified by the oxidative stress genetic score (p_interaction_ = 0.04) (Figure 1). An interquartile range increase in particle number was associated with a 0.7% (95% CI: −2.0% to 3.5%) and a 4.1% (95% CI: 1.5% to 6.8%) increase in fibrinogen, among participants with low and high oxidative stress scores, respectively. Among participants classified with high endothelial dysfunction genetic scores, we found a greater PM_2.5_ association with fibrinogen than among individuals with lower endothelial dysfunction genetic scores (p_interaction_ = 0.06) (Figure 2). We also found some effect modification by the allelic profile related to metal processing. Compared to individuals classified with lower metal processing genetic scores, among participants with higher scores we found greater associations between particle number and fibrinogen (p_interaction_ = 0.12), C-reactive protein (p_interaction_ = 0.02), and ICAM-1 (p_interaction_ = 0.08) (Figure 3). For instance, an interquartile range increase in particle number was associated with a 1.9% (95% CI: −10.0% to 15.5%) and a 19.8% (95% CI: 7.3% to 33.9%) increase in C-reactive protein, among participants classified with low and high metal processing genetic scores, respectively.

In addition, we observed significant associations between black carbon and fibrinogen only among those with high genetic score for oxidative stress, endothelial dysfunction, and impaired metal processing (Figures 1 to 3). Numerical values of the regression coefficients and their associated 95% confidence intervals are presented in Tables S7 to S9 in File S1.

### Sensitivity analysis

After adjusting for multiple comparisons using the Bonferroni correction, none of the interactions remained statistically significant.

## Discussion

We developed a new method to investigate the role of air pollution interacting with genetic variation within relevant pathways. Our genetic score approach suggests oxidative stress, endothelial function, and metal processing as biological pathways that play a role in enhancing air pollution effects on intermediary CVD-related blood markers. We observed significant greater associations between one-week exposure to particulate air pollution and fibrinogen, C-reactive protein, and ICAM-1 among elderly participants with higher allelic risk profiles, as compared to the same associations among individuals with lower allelic risk profiles.

Our results support previous findings that showed similar intermediate-term associations between air pollutants (main effects and gene-environment interactions) and markers of coagulation and inflammation [3], [5], [27]. In the same cohort, previous studies found that levels of fibrinogen, C-reactive protein, ICAM-1, and VCAM-1 were higher after days or weeks of exposure to primary and secondary pollutants [4], [23]. Peters et al. reported that the association between 5-day averages of PM_10_ and fibrinogen was modified by single-nucleotide polymorphisms (SNPs) within the fibrinogen genes [28]. Another study found some significant interactions between air pollution exposure and fibrinogen polymorphisms [27]. Madrigano et al. also observed stronger associations between black carbon exposure and VCAM-1 among participants who were GSTM1 null homozygous [5].

The greater increases in coagulation and inflammatory markers observed among subgroups with higher allelic risk profiles happened after one-week exposure to particle number and black carbon, which are traffic-related air pollutants. In Boston, black carbon originates mostly from local emissions during the morning and afternoon rush hours, as well as from transported traffic emissions [29]. We observed significant associations between black carbon and fibrinogen among participants with higher genetic score related to the three pathways of interest. This finding suggests that genetic variations related to oxidative stress, endothelial function, and metal processing play an important role in enhancing coagulation responses occurring after black carbon exposures.

In addition, while particle number was not associated with C-reactive protein in the overall sample [23], we found a positive association among the sensitive subgroup with a higher allelic risk profile related to metal processing dysfunction. Another interesting finding in this study is that the metal processing genetic score modified the association between particle number and fibrinogen, C-reactive protein, and ICAM-1, but not the effect of fine particles. Particle number concentrations are surrogates for ultrafine particles and come mostly from fresh local traffic emissions. In Boston, although secondary organic aerosols, sulfate particles, and metals constitute a large fraction of PM_2.5_ mass [30], metals also are constituents of ultrafine particles. A previous study has suggested that transition metals bound to ambient particles and their related oxidative stress response may play an important role in cardiac toxicity of particles [15]. A study suggested that either particles or metals may translocate into the bloodstream, resulting in direct toxic effects on the atherosclerotic plaque stability, vascular endothelium function, and thrombosis [16]. Our results seem plausible and consistent with this last study and add more evidence on health effects due to metal toxicity. Our findings suggest that increased coagulation and systemic inflammatory responses may occur after metals exposure and that ultrafine particles may carry more adverse metals than fine particles.

Our results also support previous studies suggesting oxidative stress as an intermediary pathway between air pollution and cardiovascular related events [6], [7], [11], [12], [31], [32]. Oxidative stress can arise from an imbalance between the production of reactive nitrogen or oxygen species (O_2_^−^, H_2_O_2_, and ONOO^−^) and a biological system's ability to promptly detoxify intermediary reactive products or simply repair the resulting damage [33]. Disturbances in the normal redox state can cause many adverse biological effects through the production of peroxides and free radicals which damage all components of the cell. For instance, oxidative stress can lead to lipid, protein, and DNA oxidation, leading to the formation of adducts which disrupt normal cellular function. Traffic emissions have also been associated with an increase in reactive oxygen species [34], as well as a diminution of antioxidant enzyme activity [35]. Oxidative stress can also initiate proinflammatory cascades, which in turn lead to activation of antioxidant proteins and substances such as catalase and glutathione. In addition, participants with genetic variants related to oxidative stress, such as heme oxygenase 1, had a higher risk of recurrent venous thromboembolism [32]. The associations we observed between traffic-related air pollutants and fibrinogen among those with higher oxidative stress genetic score support this last study. Variants within genes that mediate oxidative stress can convey susceptibility to traffic-related air pollution and enhance coagulation responses, atherosclerosis, and aging [36].

In our study, we observed a positive association between black carbon and fibrinogen only among participants with higher genetic risk profile related to endothelial function. There is some evidence that oxidative stress and impaired endothelial function are interrelated. Many of the genes implicated in diseases associated with vascular dysfunction, such as atherosclerosis, are oxidative stress-sensitive, suggesting that an imbalance in cellular oxidative stress is a critical underlying factor [37]. The most prevailing theory of impaired endothelial function is an increase in reactive oxygen species, which can impair nitric oxide (NO) production and activity. For example, Miller et al. suggested that exposure to diesel exhaust particulates leads to oxidative stress through the generation of oxygen-centered free radicals that reduce the bioavailability of endothelium-derived NO without prior interaction with the lung or vascular tissue [38]. These two interconnected mechanisms could explain why we observed associations between black carbon and fibrinogen only among individuals with high genetic risk profiles related to oxidative stress and endothelial function. The endothelium is the layer of epithelial cells that lines the cavities of the heart, blood, lymph vessels, and the serous cavities of the body. Impaired endothelial function leads to impaired control of blood pressure, inflammation, formation of new blood vessels, and blood clotting [39]. Endothelial dysfunction is considered as a key early event in the development of atherosclerosis [1]. A high endothelial dysfunction score, representing a higher allelic risk profile, may therefore identify a subset of participants having stronger coagulation responses to traffic-related air pollution.

### Limitations and strengths

This novel score approach we developed requires relevant instrumental outcomes to perform the first selection stage. The Lasso procedure returns regression coefficients that can be used to create the score. Since our outcome instruments were specific pathway-related measurements but did not represent the entire pathway, we chose to use weights equal to 1 to construct the score from the selected gene variants. Researchers can decide which weights they prefer to use based on their instrumental outcomes. We assumed equal weights for each of the gene variants chosen by the Lasso penalization. This assumption may also be a limitation, as some of the genes polymorphisms might have different functional effects and magnitude. Our defined biological pathways were not inclusive of all genes which regulate oxidative stress, endothelial dysfunction, or impaired metal processing. Therefore, we cannot definitely rule out false negative findings. We found some strong evidence of interactions between genetic-pathway and traffic-related air pollutants, which have been measured with ambient local monitors. Although this personal urban exposure approximation is likely to induce some Berkson measurement error [40], correction for measurement error may yield less biased estimates. The air pollution exposure assessment also did not take into account individual daily activities, especially whether some participants spend more time indoors versus outdoors. Traffic emission sources of organic chemicals have been associated with increased systemic inflammation [41]. Organic carbon monitoring was only available for the last few years of our study and we therefore could not address this issue. To minimize issues related to multiple testing, we focused on only one moving average and three air pollutants. Different exposure windows and pollutants may also be relevant. Our results did not remain significant after Bonferroni's correction. Even though this adjustment is very conservative, we cannot rule out false positive results. We considered a limited number of gene variants, especially for the endothelial function and metal processing pathways. Considering a larger number of variants at the first selection stage may yield more accurate scores.

This new statistical approach extends our knowledge on known and potential genetic pathways between air pollution and cardiovascular outcomes by prospectively investigating genetic pathway-environment interactions. Our score approach, selecting the gene variants by pathway and penalizing them by a Lasso procedure, enables us to reduce the set of candidate gene variants within pathway, to test for gene-air pollution interactions by pathway, and to reduce multiple comparisons since the significance examination of the interaction is based on a one degree of freedom test. Pathway analysis borrows information from correlated genes that belong to the same pathway and provides results with increased power. Our results demonstrated that the association between air pollutants and cardiovascular-related biomarkers are likely to differ according to the genetic background.

## Supporting Information

File S1 **Combined file of supporting information.**
(DOC)
