# Supplementary material for: A Novel Genetic Score Approach Using Instruments to Investigate Interactions between Pathways and Environment: Application to Air Pollution
Source: PLoS One. 2014 Apr 22;9(4):e96000. doi: 10.1371/journal.pone.0096000 (PMC3995963; doi:10.1371/journal.pone.0096000)
Supplement: File S1 — Combined file of supporting information. (DOC) [file pone.0096000.s001.doc]

**Table S1: Genetic variants related to oxidative stress**

| **rs number** | **Gene** | **Chromosome** | **Chromo-some position** | **Variation** | **Type** |
| --- | --- | --- | --- | --- | --- |
| rs2284367 | CAT | 11 | 34441118 | A/G | Intron |
| rs1001179 | CAT | 11 | |  | 34416807 | | --- | --- | | A/G | Promoter |
| rs2300181 | CAT | 11 | 34433115 | A/G | Intron |
| rs480575 | CAT | 11 | 34424222 | C/T | Intron |
| - | HMOX1 | 22 | - | Short/Long* | Promoter |
| rs2071746 | HMOX1 | 22 | 34106672 | A/T | Promoter |
| rs5995098 | HMOX1 | 22 | 34117167 | C/G | Intron |
| rs2071749 | HMOX1 | 22 | 34113413 | A/G | Intron |
| rs2071747 | HMOX1 | 22 | 34107185 | C/G | CDS**-non synonymous |
| rs1800566 | NQO1 | 16 | 68302646 | C/T | CDS**-non synonymous |
| rs1695 | GSTP1 | 11 | 67109265 | A/G | CDS**-non synonymous |
| rs1799811 | GSTP1 | 11 | Codon 114 | Ala/Val | Exon |
| rs2282679 | GC | 4 | 72827247 | A/C | Intron |
| rs1155563 | GC | 4 | 72862352 | C/T | Intron |
| rs2301022 | GCLM | 1 | 94145466 | A/G | Intron |
| rs3170633 | GCLM | 1 | 94123916 | A/G | 3’end |
| rs4147565 | GSTM1 | 1 | 110231777 | Deletion | CDS**-non synonymous |
| - | GSTT1 | 22 | 24376133 | Deletion | CDS**-non synonymous |

* Short corresponds to less than 25 GT-repeats (0: short/short, 1: short/long, 2: long/long)

** CDS: coding sequence

**Table S2**: Genetic variants related to endothelial function

| **rs number** | **Name** | **Chromosome** | **Chromo-some position** | **Variation** | **Type** |
| --- | --- | --- | --- | --- | --- |
| rs12944039 | NOS2A | 17 | 23128891 | A/G | Intron |
| rs2297516 | NOS2A | 17 | 23119857 | A/C | Intron |
| rs2072324 | NOS2A | 17 | 23141023 | A/C | Intron |
| rs2248814 | NOS2A | 17 | 23124448 | A/G | Intron |
| rs2255929 | NOS2A | 17 | 23112094 | A/T | Intron |
| rs1137933 | NOS2A | 17 | 23130059 | C/T | CDS*-non synonymous |
| rs1800779 | NOS3 | 7 | 150320876 | A/G | Intron |
| rs1799983 | NOS3 | 7 | 150327044 | G/T | CDS*-non synonymous |
| rs2010963 | VEGFA | 6 | 43846328 | C/G | 5’UTR* |

* CDS: coding sequence

** 5’UTR: Five prime untranslated region

**Table S3**: Genetic variants related to metal processing

| **rs number** | **Name** | **Chromo-some** | **Chromo-some position** | **Variation** | **Type** |
| --- | --- | --- | --- | --- | --- |
| rs224572 | SLC11A2 | 12 | 49702787 | A/G | Intron |
| rs422982 | SLC11A2 | 12 | 49692621 | A/T | Intron |
| rs12227734 | SLC11A2 | 12 | 49678590 | A/G | Intron |
| rs11837720 | SLC11A2 | 12 | 49669866 | C/G | Intron |
| rs1005559 | SLC11A2 | 12 | 49700499 | A/T | Intron |
| rs1049296 | TF | 3 | 134977044 | C/T | CDS*-non synonymous |
| rs1799945 | HFE | 6 | 26199158 | C/G | Exon |
| rs1800562 | HFE | 6 | 26201120 | A/G | Exon |
| rs1800435 | ALAD | 9 | 115193712 | C/G | CDS*-non synonymous |

* CDS: coding sequence

**Table S4**: Functions of the genes related to oxidative stress

| **Gene variants** | **Functions (provided by *GeneCards*** (Safran et al. 2010)**)** |
| --- | --- |
| **CAT** | This gene encodes catalase, a key antioxidant enzyme defending against oxidative stress. Polymorphisms in this gene have been associated with decreases in catalase activity. Catalase serves to protect cells from the toxic effects of H_2_O_2_ by converting it to water and oxygen. |
| **HMOX1** | Hemeoxygenase, an essential enzyme in heme catabolism, cleaves heme to form biliverdin, which is subsequently converted to bilirubin by biliverdin reductase, and carbon monoxide, a putative neurotransmitter. Hemeoxygenase activity is induced by its substrate heme and by various nonheme substances. |
| **NQO1** | This gene is a member of the NAD(P)H dehydrogenase (quinone) family and encodes a cytoplasmic 2-electron reductase. This protein's enzymatic activity prevents the one electron reduction of quinones that results in the production of radical species. Mutations in this gene have been associated with an increased risk of hematotoxicity after exposure to benzene. |
| **GSTP1** | Glutathione S-Transferases (GSTs) are enzymes that play an important role in detoxification by catalyzing the conjugation of many hydrophobic and electrophilic compounds with reduced glutathione. |
| **GC** | The protein encoded by this gene belongs to the albumin gene family. It binds to vitamin D and its plasma metabolites and transports them to target tissues. In plasma, it carries the vitamin D sterols and prevents polymerization of actin by binding its monomers. |
| **GCLM** | Glutamate-cysteine ligase is the first rate limiting enzyme of glutathione synthesis. The enzyme consists of two subunits, a heavy catalytic subunit and a light regulatory subunit. Gamma glutamylcysteine synthetase deficiency has been implicated in some forms of hemolytic anemia. |
| **GSTM1** | This gene encodes a GST that belongs to the mu class. Mu class enzymes function in the detoxification of electrophilic compounds, including environmental toxins and products of oxidative stress, by conjugation with glutathione. Null mutations of this gene have been linked with an increase in a number of cancers, likely due to an increased susceptibility to environmental toxins and carcinogens. |
| **GSTT1** | This gene encodes a GST that belongs to the theta class. GSTs are proteins that catalyze the conjugation of reduced glutathione to a variety of electrophilic and hydrophobic compounds. Conjugation of reduced glutathione to a wide number of exogenous and endogenous hydrophobic electrophiles. |

**Table S5: Functions of the genes related to endothelial function**

| **Gene variants** | **Functions (provided by *GeneCards*** (Safran et al. 2010)**)** |
| --- | --- |
| **NOS2A** | This gene encodes a nitric oxide synthase, which is expressed in liver and is inducible by a combination of lipopolysaccharide and certain cytokines. Produces NO which is a messenger molecule with diverse functions throughout the body. In macrophages, NO mediates tumoricidal and bactericidal actions. |
| **NOS3** | Variations in this gene are associated with susceptibility to coronary spasm. Endothelial nitric oxide synthase (eNOS), along with inducible nitric oxide synthase and neuronal nitric oxide synthase, catalyze the generation of NO and L-citrulline from L-arginine and molecular oxygen. eNOS is a critical mediator of cardiovascular homeostasis through regulation of the diameter of blood vessels. |
| **VEGF** | This gene is a member of the VEGF growth factor family and encodes a protein that specifically acts on endothelial cells and has various effects, including mediating increased vascular permeability, inducing angiogenesis, vasculogenesis and endothelial cell growth, promoting cell migration, and inhibiting apoptosis. |

**Table S6: Functions of the genes related to metal processing**

| **Gene variants** | **Functions (provided by *GeneCards*** (Safran et al. 2010)**)** |
| --- | --- |
| **SLC11A2** | This gene encodes a member of the solute carrier family 11 protein family that transports divalent metals and is involved in iron absorption. Mutations in this gene are associated with hypochromic microcytic anemia with iron overload. |
| **TF** | This gene encodes a glycoprotein that transport iron from the intestine, reticulo endothelial system, and liver parenchymal cells to all proliferating cells in the body. This protein may also have a physiologic role as granulocyte/pollen-binding protein involved in the removal of certain organic matter from serum. |
| **HFE** | The protein encoded by this gene is a membrane protein that functions to regulate iron absorption by regulating theinteraction of the transferrin receptor with transferrin. The iron storage disorder, hereditary haemochromatosis, is a recessive genetic disorder that results from defects in this gene. Binds to transferrin receptor (TFR) and reduces its affinity for iron-loaded transferring. |
| **ALAD** | ALAD catalyzes the second step in the porphyrin and heme biosynthetic pathway; zinc is essential for enzymatic activity. ALAD enzymatic activity is inhibited by lead and a defect in the ALAD structural gene can cause increased sensitivity to lead poisoning. |

**Table S7: Associations between air pollutant and blood markers according to the oxidative stress genetic score**

Fibrinogen

| **Pollutant** | **Estimate*** | **95% CI lower bound** | **95% CI upper bound** | **p-value of the interaction term** | **Oxidative stress genetic score** |
| --- | --- | --- | --- | --- | --- |
| Particle number | 0.007 | -0.020 | 0.035 | p=0.036 | Low |
| Particle number | 0.040 | 0.015 | 0.066 |  | High |
| Black carbon | 0.010 | -0.035 | 0.055 | p=0.206 | Low |
| Black carbon | 0.045 | 0.005 | 0.084 |  | High |
| PM_2.5_ | -0.008 | -0.032 | 0.016 | p=0.388 | Low |
| PM_2.5_ | 0.005 | -0.014 | 0.024 |  | High |

C-reactive protein

| **Pollutant** | **Estimate*** | **95% CI lower bound** | **95% CI upper bound** | **p-value of the interaction term** | **Oxidative stress genetic score** |
| --- | --- | --- | --- | --- | --- |
| Particle number | 0.110 | -0.020 | 0.239 | p=0.427 | Low |
| Particle number | 0.052 | -0.069 | 0.174 |  | High |
| Black carbon | -0.023 | -0.199 | 0.154 | p=0.907 | Low |
| Black carbon | -0.010 | -0.160 | 0.140 |  | High |
| PM_2.5_ | 0.065 | -0.039 | 0.169 | p=0.161 | Low |
| PM_2.5_ | -0.025 | -0.108 | 0.059 |  | High |

ICAM-1

| **Pollutant** | **Estimate*** | **95% CI lower bound** | **95% CI upper bound** | **p-value of the interaction term** | **Oxidative stress genetic score** |
| --- | --- | --- | --- | --- | --- |
| Particle number | 0.095 | 0.070 | 0.121 | p=0.519 | Low |
| Particle number | 0.087 | 0.063 | 0.110 |  | High |
| Black carbon | 0.047 | 0.007 | 0.088 | p=0.076 | Low |
| Black carbon | 0.004 | -0.030 | 0.038 |  | High |
| PM_2.5_ | 0.061 | 0.037 | 0.084 | p=0.557 | Low |
| PM_2.5_ | 0.052 | 0.034 | 0.071 |  | High |

VCAM-1

| **Pollutant** | **Estimate*** | **95% CI lower bound** | **95% CI upper bound** | **p-value of the interaction term** | **Oxidative stress genetic score** |
| --- | --- | --- | --- | --- | --- |
| Particle number | 0.098 | 0.064 | 0.133 | p=0.559 | Low |
| Particle number | 0.087 | 0.055 | 0.119 |  | High |
| Black carbon | 0.035 | -0.015 | 0.085 | p=0.334 | Low |
| Black carbon | 0.006 | -0.035 | 0.048 |  | High |
| PM_2.5_ | 0.044 | 0.015 | 0.073 | p=0.451 | Low |
| PM_2.5_ | 0.030 | 0.007 | 0.053 |  | High |

* regression coefficient for an interquartile increase in air pollution

**Table S8: Associations between air pollutant and blood markers according to the endothelial dysfunction genetic score**

Fibrinogen

| **Pollutant** | **Estimate*** | **95% CI lower bound** | **95% CI upper bound** | **p-value of the interaction term** | **Endothelial dysfunction genetic score** |
| --- | --- | --- | --- | --- | --- |
| Particle number | 0.043 | 0.013 | 0.074 | p=0.280 | Low |
| Particle number | 0.026 | 0.003 | 0.048 |  | High |
| Black carbon | 0.006 | -0.046 | 0.057 | p=0.239 | Low |
| Black carbon | 0.040 | 0.006 | 0.074 |  | High |
| PM_2.5_ | -0.018 | -0.043 | 0.008 | p=0.064 | Low |
| PM_2.5_ | 0.010 | -0.008 | 0.027 |  | High |

C-reactive protein

| **Pollutant** | **Estimate*** | **95% CI lower bound** | **95% CI upper bound** | **p-value of the interaction term** | **Endothelial dysfunction genetic score** |
| --- | --- | --- | --- | --- | --- |
| Particle number | 0.056 | -0.093 | 0.204 | p=0.642 | Low |
| Particle number | 0.092 | -0.013 | 0.196 |  | High |
| Black carbon | 0.063 | -0.136 | 0.263 | p=0.363 | Low |
| Black carbon | -0.037 | -0.165 | 0.091 |  | High |
| PM_2.5_ | 0.031 | -0.078 | 0.141 | p=0.667 | Low |
| PM_2.5_ | 0.004 | -0.069 | 0.077 |  | High |

ICAM-1

| **Pollutant** | **Estimate*** | **95% CI lower bound** | **95% CI upper bound** | **p-value of the interaction term** | **Endothelial dysfunction genetic score** |
| --- | --- | --- | --- | --- | --- |
| Particle number | 0.085 | 0.057 | 0.113 | p=0.775 | Low |
| Particle number | 0.089 | 0.068 | 0.110 |  | High |
| Black carbon | 0.073 | 0.028 | 0.118 | p=0.002 | Low |
| Black carbon | -0.006 | -0.036 | 0.023 |  | High |
| PM_2.5_ | 0.077 | 0.052 | 0.101 | p=0.016 | Low |
| PM_2.5_ | 0.043 | 0.026 | 0.060 |  | High |

VCAM-1

| **Pollutant** | **Estimate*** | **95% CI lower bound** | **95% CI upper bound** | **p-value of the interaction term** | **Endothelial dysfunction genetic score** |
| --- | --- | --- | --- | --- | --- |
| Particle number | 0.069 | 0.031 | 0.108 | p=0.242 | Low |
| Particle number | 0.093 | 0.065 | 0.121 |  | High |
| Black carbon | 0.040 | -0.015 | 0.095 | p=0.352 | Low |
| Black carbon | 0.012 | -0.025 | 0.048 |  | High |
| PM_2.5_ | 0.048 | 0.018 | 0.078 | p=0.372 | Low |
| PM_2.5_ | 0.032 | 0.012 | 0.053 |  | High |

* regression coefficient for an interquartile increase in air pollution

**Table S9: Associations between air pollutant and blood markers according to the metal processing dysfunction genetic score**

Fibrinogen

| **Pollutant** | **Estimate*** | **95% CI lower bound** | **95% CI upper bound** | **p-value of the interaction term** | **Metal processing dysfunction genetic score** |
| --- | --- | --- | --- | --- | --- |
| Particle number | 0.020 | -0.008 | 0.047 | p=0.120 | Low |
| Particle number | 0.043 | 0.019 | 0.067 |  | High |
| Black carbon | 0.022 | -0.023 | 0.066 | p=0.337 | Low |
| Black carbon | 0.048 | 0.010 | 0.085 |  | High |
| PM_2.5_ | 0.002 | -0.020 | 0.024 | p=0.814 | Low |
| PM_2.5_ | 0.005 | -0.013 | 0.024 |  | High |

C-reactive protein

| **Pollutant** | **Estimate*** | **95% CI lower bound** | **95% CI upper bound** | **p-value of the interaction term** | **Metal processing dysfunction genetic score** |
| --- | --- | --- | --- | --- | --- |
| Particle number | 0.019 | -0.105 | 0.144 | p=0.017 | Low |
| Particle number | 0.181 | 0.070 | 0.292 |  | High |
| Black carbon | 0.041 | -0.125 | 0.207 | p=0.910 | Low |
| Black carbon | 0.030 | -0.110 | 0.170 |  | High |
| PM_2.5_ | -0.002 | -0.098 | 0.094 | p=0.460 | Low |
| PM_2.5_ | 0.042 | -0.035 | 0.119 |  | High |

ICAM-1

| **Pollutant** | **Estimate*** | **95% CI lower bound** | **95% CI upper bound** | **p-value of the interaction term** | **Metal processing dysfunction genetic score** |
| --- | --- | --- | --- | --- | --- |
| Particle number | 0.073 | 0.049 | 0.097 | p=0.076 | Low |
| Particle number | 0.096 | 0.074 | 0.117 |  | High |
| Black carbon | 0.011 | -0.027 | 0.048 | p=0.738 | Low |
| Black carbon | 0.018 | -0.014 | 0.050 |  | High |
| PM_2.5_ | 0.049 | 0.027 | 0.071 | p=0.556 | Low |
| PM_2.5_ | 0.057 | 0.040 | 0.074 |  | High |

VCAM-1

| **Pollutant** | **Estimate*** | **95% CI lower bound** | **95% CI upper bound** | **p-value of the interaction term** | **Metal processing dysfunction genetic score** |
| --- | --- | --- | --- | --- | --- |
| Particle number | 0.091 | 0.057 | 0.124 | p=0.944 | Low |
| Particle number | 0.089 | 0.060 | 0.119 |  | High |
| Black carbon | 0.033 | -0.013 | 0.080 | p=0.623 | Low |
| Black carbon | 0.020 | -0.019 | 0.059 |  | High |
| PM_2.5_ | 0.047 | 0.020 | 0.074 | p=0.450 | Low |
| PM_2.5_ | 0.035 | 0.013 | 0.056 |  | High |

* regression coefficient for an interquartile increase in air pollution
